# The “coordination conjecture” as an alternative to Patel’s fortuitous enhancement hypothesis for the relation between vocal learning and beat-based dancing

**DOI:** 10.1186/s12868-024-00868-x

**Published:** 2024-11-06

**Authors:** Gregory Hickok

**Affiliations:** Departments of Cognitive Sciences and Language Science, University of California, Irvine, CA USA

## Abstract

Patel proposes a viable hypothesis regarding the relation between vocal learning and beat-based dancing but it is not without problems. I highlight these problems and propose a solution, the “coordination conjecture.”

I agree with Patel [1] that the unique parallels between humans and parrots in terms of their capacity for vocal learning and their ability to synchronize motorically to an auditory rhythm—beat perception and synchronization (BPS)—suggest a possible connection. I also agree that BPS emerged as a fortuitous trait (a *spandrel*) in parrots, where it remains so, as well as in humans, where it may or may not have undergone further selection. And I further agree with Patel on two additional points, (i) that what distinguishes parrots and humans from other vocal learners, like songbirds who don’t exhibit BPS, is that parrots and humans have a more complex vocal learning system, and (ii) that this complexity is related to the added ability of parrots and humans to control supra-syringeal/laryngeal vocal organs, which seems to involve a dual neural control system in both species [2, 3].

Patel’s proposal differs from my own views, however, in terms of *why* adding this more complex, dual vocal control system results in BPS. In the context of the human system, Patel argues that the increase in complexity drove stronger auditory integration with the dorsal premotor pitch control system, which fortuitously strengthened an indirect pathway to nearby nonvocal motor systems via the angular gyrus. In his words,

…the evolution of strong integration between auditory regions and vocal dorsal premotor regions in ancestral humans (via the laryngeal pitch control pathway) involved gene regulation changes which fortuitously enhanced the strength of neural connections between auditory and nonvocal dorsal premotor regions near the vocal dorsal premotor regions.

There are three related problems with this hypothesis.

First, there is no explanation for why an evolutionary change that is adaptive for strengthened auditory-motor control of vocal effectors should involve nonvocal motor systems (directly or indirectly via the angular gyrus). This is not evidence *against* Patel’s hypothesis as there are many unknowns in any theory, but the second problem deepens the concern.

Second, my colleagues and I have argued that the dorsal laryngeal pitch control pathway evolved prior to the more ventral phonetic/syllabic supralaryngeal control pathway and is analogous to what is found in songbirds, i.e., an auditory-motor integration circuit for controlling pitch-related vocalization via the larynx/syrinx [2]. The evolution of this dorsal laryngeal pitch control pathway presumably involved some kind of strengthening in auditory-motor integration in humans, parrots, and songbirds. So, it appears that the evolution of strengthened auditory-motor integration for (let’s call it) “level 1” vocal learning does not *necessarily* result in enhanced connectivity to nonvocal motor systems, otherwise songbirds should have BPS. This implies that enhanced connectivity to nonvocal motor systems must result from enhanced “level 2” vocal learning, like that found only in parrots and humans. The problem, then, is why “level 2” vocal learning should lead to connectivity with nonvocal motor systems (e.g., between auditory cortex and the angular gyrus) but “level 1” does not. As far as I can tell, Patel does not have an answer, which is why he terms it “fortuitous”. This problem deepens concern for the first one because now we need an explanation for why evolutionary changes that strengthen auditory-motor integration involve nonvocal motor systems in some cases but not others.

Third, as noted, Patel and I agree that the increase in complexity of the parrot and human neural architecture for controling vocalization is a consequence of the use of independent vocal articulators. He further argues that this requires strengthening of auditory-motor connectivity to a level beyond that observed in songbirds (i.e., “level 2”). Yet, Patel assumes that this neural strengthening was applied to the dorsal laryngeal pitch control pathway (see the quote above). This raises a problem: why does the dorsal laryngeal pitch control pathway need auditory-motor strengthening when a nonlaryngeal control system is what is added in “level 2” vocal learning?

As Patel pointed out, my colleagues and I offered a brief conjecture on the origin of BPS that provides an alternative to Patel’s. We suggested that “rhythmic synchronization, the ability to synchronize movement to an auditory beat… is a necessary function enabling the coordination of the two proposed [vocal control] streams” (p. 1785) [2]. This general idea, which I will elaborate on shortly, offers possible solutions to the three problems with Patel’s hypothesis.

1) *Why do evolutionary changes related to level 2 vocal learning involve spreading of auditory connectivity to multiple systems?* Because level 2 vocal learning necessarily involves the synchronization of multiple motor systems to the same auditory rhythm.

2) *Why doesn’t level 1 auditory-motor strengthening result in nonvocal spreading?* Because it only involves auditory control of one motor effector system.

3) *Why does adding a supralaryngeal vocal motor system result in strengthened auditory-laryngeal control?* It doesn’t. It involves expanding auditory control of vocal effectors to include supralaryngeal motor systems.

For these reasons, I find the “coordination conjecture” for BPS attractive. But in order to raise the idea above a conjecture, we need to answer an important question: why is BPS a necessary function for coordinating laryngeal and supralaryngeal vocal motor systems? My colleagues and I did not attempt to answer this question. In what follows, I develop the argument in a bit more detail.

To start, I would like to suggest that our conjecture stated things rather backwards. Rather than beat synchronization being a solution to what’s needed for speech coordination, I think it’s more accurate to say that beat synchronization is a simple version of what’s required for speech coordination. So, the idea is that if you build a multi-effector coordination system for speech, you get BPS for free. Let me explain.

## The need for temporal coordination and prediction in speech planning

Modulation of pitch during speech, a component of prosody or intonation, occurs over different levels of linguistic organization, such as the phrase, word, or syllable. The temporal coordination of pitch features at all of these levels has important effects on the communicative content of an utterance, indicating, for example, distinctions between questions and statements, phrasal boundaries, lexical contrasts (e.g., in tonal languages), and topic focus (e.g., pitch accents on different words signal different meanings as in *I THOUGHT he left* vs. *I thought HE left*). Thus, it is important to coordinate the timing of laryngeal pitch control with supralaryngeal phonetic/syllabic control to ensure that pitch modulations occur in the correct place in the sequence of syllables. Research on the timing of such coordination has indicated rather precise synchronization, particularly between syllable *boundaries* and pitch features such as lexical tone or pitch accent [4, 5]. Moreover, this work has shown that planning for coordinated articulation requires a form of prediction—an important feature of BPS highlighted by Patel—because generating changes in voice pitch during speech is not instantaneous and in fact is rather slow, taking approximately 100 msec to traverse a 4 semitone change in pitch [6]. This means that in order to time a pitch peak or valley to a syllable boundary, pitch change toward that target needs to be initiated more than 100 msec earlier.

## The problem of quasi-rhythmic speech

If speech rhythms pulsed at a regular isochronous beat, i.e., more similar to musical rhythms, solving the dual system coordination problem might be relatively easy. As Patel points out, “The motor system is adept at generating periodic movements on the timescale of beats”. In principle, then, two motor control systems could simply align themselves separately to some kind of internal motor-centric metronome. But speech is only quasi-periodic. To be sure, speech does have an *average* rhythm, detectible in the amplitude envelope (basically tracking the syllable rate) of natural language [7]. But one cannot use an average rhythm to generate a precise prediction of the onset of the next syllable in any given utterance. To get an intuitive sense of the quasi-rhythmic speech problem, clap along with the syllables as you read the following sentence aloud. *Juan wrote a rough draft and then he edited it.* This is why we don’t dance to conversational speech; the rhythm is not regular or predictable enough to the listener [8].

So, the fact that the motor system is adept at generating rhythmic movements (such as walking, jumping, clapping, tapping, chest thumping, chewing) is not particularly helpful in coordinating laryngeal and supralaryngeal vocal effectors for the purpose of generating natural speech. What’s needed is a way to coordinate these separate subsystems so that they align to an utterance-dependent quasi-rhythm that *breaks* with the natural tendency of the motor system to generate rhythmic regularity.

## Towards a quasi-rhythmic beat perception and synchronization (QR-BPS) system

While there is extensive research regarding the perception of, and synchronization to, predictable rhythmic patterns, relatively little attention has been paid to how less predictable rhythms are processed. This is not surprising, at least for overt motor synchronization (like tapping to the beat) because tapping to an unpredictable sequence of pulses equates to a simple reaction time task. The perceptual side is potentially informative, however, because it can answer the question of whether unpredictable pulse sequences can be learned and remembered efficiently. If the answer is yes, then we have a candidate basis for synchronizing independent motor systems to a quasi-rhythm, i.e., a neural code for the target quasi-rhythm that can be used to plan the timing of motor commands in the two systems. Put differently, a temporal pattern of pulses can be predictable in more than one way. It can follow a regular pattern (incoming beats follow the pattern of previous beats) or it can follow a previously learned template for an irregular (or quasi-rhythmic) pattern.

Can humans learn irregular temporal patterns efficiently? Apparently, yes. Kang and colleagues [9] presented listeners with temporal patterns comprised of random sequences of irregularly spaced clicks and asked them to detect repetitions of these sequences, indicating perceptual learning. They report that listeners were able to learn these patterns rapidly after only a few exposures and did so over a broad range of inter-click intervals, from a sparse 5 clicks per second to a dense 50 clicks per second. Moreover, learning was also shown to occur implicitly over the course of the session for sequences that were repeated occasionally, interspersed among other unique stimuli during testing. This *perceptual* ability to acoustically code irregular rhythms—as opposed to the ability to motorically synchronize to rhythm—does *not* appear to be unique to humans [10], arguing that (QR-)BPS in humans does not result from a specialized auditory system, but rather a unique sensorimotor connectivity pattern, a point of agreement with Patel. Given that the auditory system is capable of learning even random temporal patterns, it should not be surprising that rhythms in some musical traditions, e.g., Malian djembe, make use of more irregular rhythmic patterns [11].

It seems, then, that we have a possible foundation for supporting a component of QR-BPS: an auditory system capable of learning and storing irregular temporal patterns that could serve as the targets for coordinated action across multiple motor subsystems, assuming appropriate connectivity. The idea that a quasi-rhythmic or random temporal pattern can be stored in the auditory system and serve as a target for action that is synchronized with that pattern is a testable hypothesis: it predicts that tapping to a learned, quasi-speech-like rhythm or *random* beat pattern would show similar^1^ characteristics to what is reported for BPS experiments with regular rhythms in humans.

But how would this basic ability translate to speech? Surely, the rhythmic patterns we generate during speaking are not called up from a great store of previously learned patterns covering all possible sequences of syllables, stress patterns, and rates of speech. No, like other aspects of linguistic structure, prosodic planning and the rhythms it contains are part of a generative system that is integrated with the generation of segmental/phonemic and morphosyntactic patterns [12]. One consequence of such planning (i.e., a level of representation generated) may be a quasi-rhythm pattern coded in the auditory system that could serve as the target for motor coordination.

Although this idea is speculative so far, there is some evidence to support the existence of an auditory rhythmic code playing a role in speech production. It comes from research on delayed auditory feedback, which disrupts speech fluency and tends to do so most dramatically at a delay interval of 200 msec. The standard assumption is that the delay causes interference at the segmental/syllabic phonological level: the system expected to hear syllable *S* in the feedback but heard *S-1* (the previous syllable) instead. Howell et al. [13] proposed a *rhythm-based* alternative account to the standard phonemic *content* model, noting that 200 msec is approximately the duration of a syllable and that speech delayed at this interval would cause mismatches in expected versus perceived rhythmic stress patterns. Kaspar and Rübeling [14] tested this hypothesis directly by asking participants to read repeated sequences of syllable pairs that varied in two dimensions, phonemic content (same syllable: tata tata tata… vs. different syllables: tali tali tali…) and stress pattern (uniform stress: tata tata tata… vs. accented stress: TAta TAta TAta) under conditions of delated auditory feedback. Note that for the different syllable and accented stress stimuli, 200 msec feedback delays would result in a phase shift of phonemic content (tali->lita) or stress pattern (TAta ->taTA). They report that phonemic content mismatches (tali relative to tata) had no effect on reading times whereas rhythmic mismatches did (TAta/TAli relative to tata/tali). This demonstrates the influence of some sort of auditory rhythmic code during speech production planning and fits nicely with recent proposals that prosodic features provide a planning frame for speech production [15]. Interestingly, delayed auditory feedback seems to drive activity in the dorsal, laryngeal pitch-related pathway more strongly and faster than the ventral pathway [16].

Together these arguments establish the feasibility of auditory-related rhythmic/prosodic representations serving as targets for coordinating the multiple motor systems involved in speech. To summarize, the problem is how to temporally coordinate pitch features with phonetic/syllabic boundaries during vocalization, which has been shown to be quite precise even though the timing is only quasi-rhythmic and varies from utterance to utterance. The solution that I am proposing is that speech planning involves the integration of prosodic features and phonetic/syllabic sequences that generates an auditory-based representation of the quasi-rhythmic target, which is used as a temporal reference to synchronize articulatory planning of separately controlled effectors. Once the system has the ability to generate an auditory-based target rhythm and, crucially, to wire it up to motor systems for the purpose of synchronizing to it, an externally provided and *predictable* rhythm would then function as a (simpler) target for motor synchronization, i.e., BPS emerges for free.

These sorts of arguments are more easily made in the case of humans than parrots, where less is known (as far as I’m aware). But one prediction of my hypothesis is that parrots should also exhibit vocal abilities that require some degree of temporally precise coordination between syringeal and suprasyringeal effectors.

## Why does BPS involve nonvocal systems?

Patel suggests that BPS spreads to nonvocal motor systems simply as a (unexplained) fortuitous consequence of level 2 auditory-laryngeal system strengthening, implemented anatomically via an indirect pathway from auditory-cortex to the angular gyrus to nonvocal dorsal premotor areas. The idea that BPS results from the need to coordinate independent vocal articulators gets us one step closer to an explanation because synchronizing more than one effector system to a (quasi-)rhythm *is* the computational problem that level 2 vocal learning solved. In short, some degree of (QR-)BPS spreading is a computational requirement for speech. But it doesn’t get us all the way because, logically speaking, (QR-)BPS didn’t have to spread to nonvocal systems for speech to emerge.

I don’t think we have an answer yet, but I can see at least one avenue to explore in the search for a solution. At the most general level, perhaps it’s easier to spread (QR-)BPS to the whole of the motor system than to just two motor planning pathways. It may be relevant that the human dorsal laryngeal pathway is more strongly auditory-weighted than the ventral phonetic/syllabic pathway [17] hinting that the neural architecture of (QR-)BPS spreading did not involve the evolution of a duplicate, parallel auditory-motor circuit into the ventral phonetic/syllabic pathway (otherwise both should be strongly auditory-weighted), which isn’t consistent with pan-motor (QR-)BPS spreading anyway. It seems more likely that multi-effector synchronization was achieved by patching into a system that has broader communication with multiple motor subsystems such as circuits involving the basal ganglia, supplementary motor complex, and cerebellum all of which have been implicated in BPS, as Patel notes. Perhaps it’s relevant that a particular zone of the cerebellum has been implicated in ataxic dysarthria, a motor speech disorder involving dyscoordination during the articulation of individual speech gestures (consonants and vowels) as well as a tendency to produce isochronously timed syllables rather than the normal quasi-rhythmic timing [18]. Preliminary analyses in my lab suggest functional connectivity of this cerebellar region to the SMA and the dorsal and ventral precentral speech areas.

## Data Availability

N/A.

## Footnotes

1 I say similar because, in general, we might expect better performance on regular rhythms compared to irregular rhythms simply as a function of task difficulty. This was true in part for the Kang, et al. experiment.
